# Bayesian statistical modeling to predict observer-specific optimal windowing parameters in magnetic resonance imaging

**DOI:** 10.1016/j.heliyon.2023.e19038

**Published:** 2023-08-08

**Authors:** Kohei Sugimoto, Masataka Oita, Masahiro Kuroda

**Affiliations:** a Graduate School of Interdisciplinary Science and Engineering in Health Systems, Okayama University, 5-1 Shikata-cho, 2-chome, Kita-ku, Okayama, Okayama, 700-8558, Japan; b Division of Imaging Technology, Okayama Diagnostic Imaging Center, 3-25, Daiku, 2-chome, Kita-ku, Okayama, Okayama, 700-0913, Japan; c Faculty of Interdisciplinary Science and Engineering in Health Systems, Okayama University, 5-1 Shikata-cho, 2-chome, Kita-ku, Okayama, Okayama, 700-8558, Japan; d Department of Radiological Technology, Faculty of Health Sciences, Okayama University, 5-1 Shikata-cho, 2-chome, Kita-ku, Okayama, Okayama, 700-8558, Japan

**Keywords:** MR image, Image intensity standardization, Windowing, Prediction, Bayesian statistical modeling

## Abstract

Magnetic resonance (MR) images require a process known as windowing for optimizing the display conditions. However, the conventional windowing process often fails to achieve the preferred display conditions for observers due to various factors. This study proposes a novel framework for predicting the preferred windowing parameters for each observer using Bayesian statistical modeling. MR images obtained from 1000 patients were divided into training and test sets at a 7:3 ratio. The image intensity and windowing parameters were standardized using previously reported methods. Bayesian statistical modeling was utilized to predict the windowing parameters preferred by three MR imaging (MRI) operators. The performance of the proposed framework was evaluated by assessing the mean relative error (MRE), mean absolute error (MAE), and Pearson's correlation coefficient (ρ) of the test set. In addition, the naive method, which presumes that the average value of the windowing parameters for each acquisition sequence and body region in the training set is optimal, was also used for comparison. Three MRI operators and three radiologists conducted visual assessments. The mean MRE, MAE, and ***ρ*** values for the window level and width (WL/WW) in the proposed framework were 12.6 and 13.9, 42.9 and 85.4, and 0.98 and 0.98, respectively. These results outperformed those obtained using the naive method. The visual assessments revealed no significant differences between the original and predicted display conditions, indicating that the proposed framework accurately predicts individualized windowing parameters with the additional advantages of robustness and ease of use. Thus, the proposed framework can effectively predict the windowing parameters preferred by each observer.

## Highlights

• A Bayesian statistical model was developed to predict the preferred WL/WW.
• Image intensity standardization was applied to create a statistical model.
• A model was created easily from the existing data without additional training.

## 1 Introduction

As magnetic resonance (MR) images have a data depth of 12-bit, the conversion of the data depth to 8-bit is necessary to display the images on a monitor. This process is known as “windowing,” and it is accomplished by adjusting the window level and window width (WL/WW) [1]. In clinical practice, the MR imaging (MRI) operator subjectively adjusts these parameters according to individual preferences; however, these values are often unstable as they depend on the observer and viewing conditions.

Several neural network (NN) models have been proposed to objectively and automatically estimate the optimal windowing parameters (Table 1). A three-layer NN was used to automatically adjust the WL/WW for MR images of the brain in a previous study [2]. A recent study [3] demonstrated that a convolutional NN (CNN) model could estimate the optimal WL/WW (oWL/oWW) for multiple sequences and body regions with high precision. These NN models [2,3] assumed that the windowing parameters of each MR image have a single optimal value. However, as the oWL/oWW varies depending on various factors, these NN models require multiple training data sets to predict the optimal value for each condition. Thus, creating a model is a laborious task, making its implementation in clinical practice impractical. Lai et al. [4,5] proposed an adaptive hierarchical NN model that can estimate the oWL/oWW according to personal preferences and viewing conditions via online training. However, this model [4,5] requires manual retuning by the observer, which limits its feasibility.

**Table 1:** Previous research on the predicting of the optimal windowing parameters for MRI.

Reference	Model	Number of training data	Method for creating the training set
Ohhashi et al. (1991) [2]	Shallow neural network	49, 25	adjusted by a human expert
Lai et al. (2000) [4]	Hierarchical neural network	>2400	adjusted by the human experts
Lai et al. (2005) [5]	Adaptive hierarchical neural network	2436	adjusted by the human experts
Zhao et al. (2019) [3]	Deep convolutional neural network	12715	annotated by 17 clinicians from three different hospitals.

Abbreviation: MRI, magnetic resonance imaging.

Methods for converting the signal intensity of MR images to 8-bit include windowing for visualization and signal intensity standardization in the context of image processing. Various methods have been proposed for the standardization of MR images of the brain, such as histogram matching [[6], [7], [8]], WhiteStripe [9], RAVEL [10], ComBat [[11], [12], [13]], and mica [14]. Signal intensity standardization has become a common preprocessing step in radiomics [[15], [16], [17], [18]], machine learning [15,17,[19], [20], [21]], and deep learning [[19], [20], [21], [22], [23]] in recent years. However, these signal intensity standardization methods aim to correct for site- and scanner-induced variability (scanner effects) as well as within-subject variability (intensity unit effects) [14] caused by arbitrary intensity units. Consequently, their primary purpose is not to enable visualization or windowing to achieve the preferred viewing conditions for observers. Thus, the two trends in converting the signal intensity distribution of MR images have developed separately. Although numerous reports have discussed signal intensity standardization, especially in the context of image processing, few reports have discussed adjusting the number of tones for optimal human observation. Therefore, the optimization of the display conditions of MR image is yet to be realized in clinical practice.

This study proposes a framework that applies Bayesian statistical modeling to predict the oWL/oWW for each observer and viewing condition simply and robustly. In contrast to previous methods that assumed single optimal values, this proposed framework predicts the preferred viewing conditions for each observer using a statistical model that takes into consideration the variance of oWL/oWW. Furthermore, the framework utilizes the data of the WL/WW set extracted from clinical practice to simplify the preparation of the training data. This framework represents a significant advancement over existing methods by combining simplicity of use, adaptability to individual preferences and viewing conditions, and reduced dependence on exhaustive training datasets. The oWL/oWW predicted by the proposed framework was evaluated quantitatively and qualitatively in this study to verify its capability.

## 2 Material and methods

**Fig. 1:**
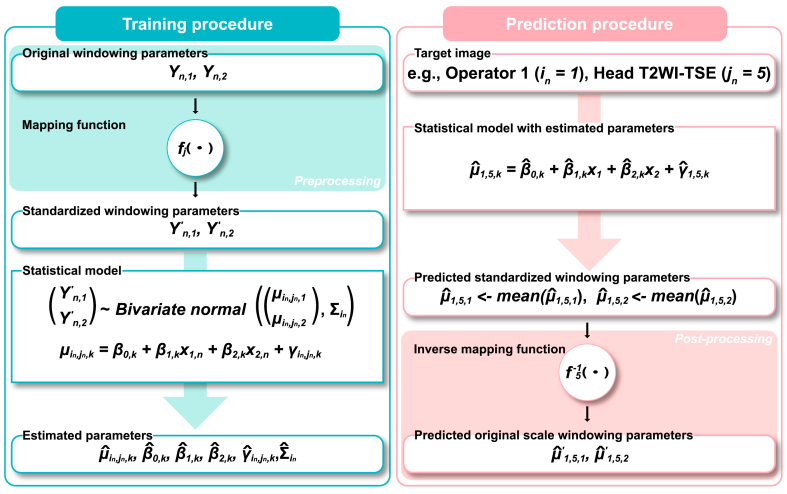
Diagram illustrating the training and prediction process for the proposed framework.

An overview of the framework is presented in Fig. 1, with further details given below.

### 2.1 Data

This study was approved by the Institutional Ethics Committee (KEN2110-007). The MR images of 1000 patients who underwent MR examinations between 2015 and 2018 were randomly selected and used in this study. All images were obtained using a 1.5 T MRI (Avanto fit; Siemens Healthineers, Erlangen, Germany). Three MRI operators adjusted the WL/WW for the MR images in clinical practice, which were defined as the ground truth of the optimal values for each MRI operator, acquisition sequence, and body region. The data were assigned to the training and test sets at a 7:3 ratio. Data from the same patient were included in the same set.

### 2.2 Preprocessing

Standardization of the WL/WW in the training set was performed by applying the image intensity standardization method as a preprocessing step [6,7]. The proposed method for standardizing the windowing parameters comprised the same training and customized transformation steps as the previously reported method [6,7]. Although the proposed method is explained in the following sections, the underlying method is described only briefly here. Preprocessing was performed based on a publicly available program used in a previous report using Python (ver. 3.8.12; Python Software Foundation, DE, USA) [24].

Standard scales were computed in the training step by averaging the intensity values of the predefined landmarks in the intensity of interest (IOI) of each MR image set for a specific acquisition sequence and body region. In this study, IOI was defined as voxels with a signal intensity higher than zero. The landmarks were used as image intensity values at the 1st, 10th, 20th, 30th, 40th, 50th, 60th, 70th, 80th, 90th, and 99th percentiles of the IOI of the image, as these landmarks showed good results [8]. The standard scales had predefined values corresponding to the minimum and maximum landmark values set to 1 and 100, respectively.

**Fig. 2:**
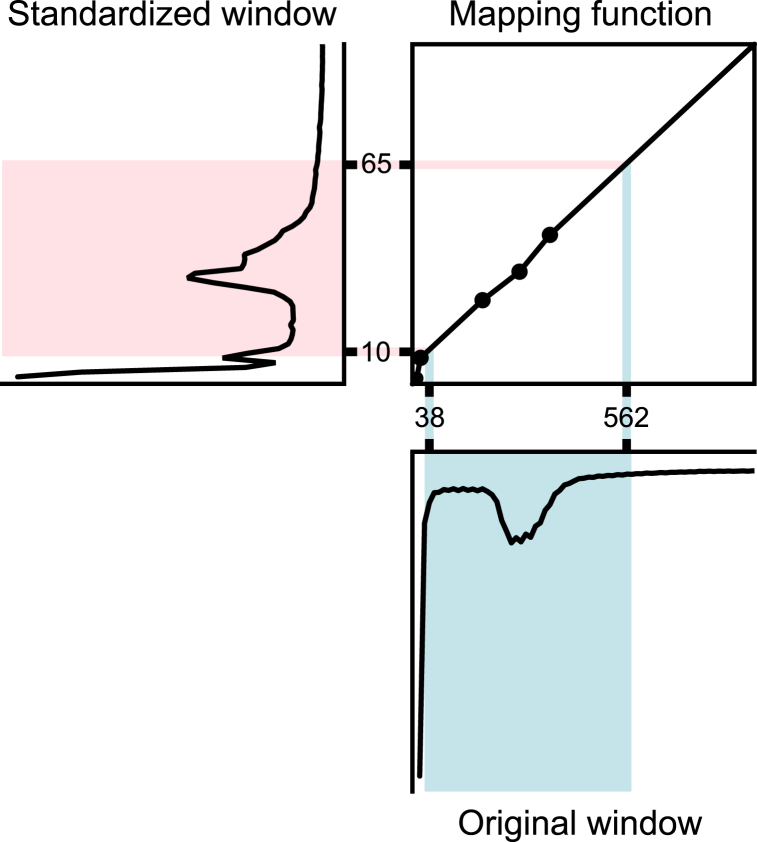
Diagram of the standardization method for the window level and width (WL/WW using the mapping function. The histogram of the input image and the lower and upper limits of its window are shown in the lower right image. These are inputs to the mapping function corresponding to the input image shown in the upper right image. As a result, the lower and upper limits of the window after standardization corresponding to the input window are output, as shown in the upper left image.

The upper and lower window limits (UW/LW) of the input images were mapped to the standard scale using the mapping function in the customized transformation step (Fig. 2). This function was created to map the signal intensity of the landmarks in the IOI of the input image on the standard scale corresponding to the acquisition sequence and body region for that image in a piecewise linear fashion. The UW/LW was calculated from WL/WW using equations (1), (2):

(1) UW=WL+WW/2,

(2) LW=WL−WW/2

The standardized UW/LW (sUW/sLW) output from the customized transformation step was subsequently converted to standardized WL/WW (sWL/sWW) using equations (3), (4):

(3) sWW=sUW−sLW,

(4) sWL=sLW+sWW/2.

### 2.3 Statistical model

A Bayesian statistical model was fitted to predict the oWL/oWW using the training set with the aforementioned preprocessing steps. The Bayesian statistical model infers the parameters as a posterior distribution from the likelihood and prior distribution according to Bayes' rule, as described by equation (5):

(5) p(θ|y)∝p(y|θ)p(θ)

where p(θ|y) is the posterior distribution, p(y|θ) is the likelihood, and p(θ) is the prior distribution. y and θ represent the data and parameters, respectively. The assumptions of the model were as follows:

$Y_{n,1}$ and $Y_{n,2}$ denote the sets of sWL and sWW at index n(n=1,…,N) in the training set after preprocessing, respectively, and are assumed to follow the bivariate normal distribution, as shown in equation (6):

(6) $$\left(\begin{array}{c} Y_{n,1}\\ Y_{n,2} \end{array}\right)∼Bivariate\;normal\left(\left(\begin{array}{c} \mu_{i_{n},j_{n},1}\\ \mu_{i_{n},j_{n},2} \end{array}\right),\Sigma_{i_{n}}\right)$$

where $i_{n}\;\left(i_{n}=1,2,3\right)$ and $j_{n}\;\left(j_{n}=1,\ldots ,J\right)$ represent the MRI operator and imaging condition (that is, the combinations of the acquisition sequences and body regions) when the index is n, respectively. J represents the number of imaging conditions used. $\left(\begin{array}{c} \mu_{i_{n},j_{n},1}\\ \mu_{i_{n},j_{n},2} \end{array}\right)$ is the mean vector and $\Sigma_{i_{n}}$ is the covariance matrix for the MRI operator $i_{n}$.

Subsequently, a mixed-effect model was assumed for $\mu_{i_{n},j_{n},k}\;\left(k=1,2\right)$, in which the difference between the MRI operators was a fixed effect $\beta =\left\{\beta_{0,k},\beta_{1,k},\beta_{2,k}\right\}$, and the difference between the imaging conditions was a random effect $\gamma_{i_{n},j_{n},k}$. This difference is expressed in equation (7):

(7) $$\mu_{i_{n},j_{n},k}=\beta_{0,k}+\beta_{1,k}x_{1,n}+\beta_{2,k}x_{2,n}+\gamma_{i_{n},j_{n},k}\text{,}$$

where $x_{1,n}$ and $x_{2,n}$ are dummy variables. $\gamma_{i_{n},j_{n},k}$ is assumed to follow a normal distribution with a mean of 0 and a standard deviation of $\tau_{j_{n}}$. All parameters were assumed to follow a non-informative prior distribution.

### 2.4 Estimation

A joint posterior distribution was constructed based on the abovementioned assumptions, and the parameters were estimated using the training set after preprocessing. The parameters were estimated using Bayesian inference with the Markov chain Monte Carlo (MCMC) method utilizing the Hamiltonian Monte Carlo algorithm. R (ver. 4.1.3; R Foundation, Vienna, Austria) and RStan package (ver. 2.19.3) were used to compute the MCMC samples. The MCMC conditions were as follows: number of chains, 4; thinning of chains, 5; warm-up iteration number, 1000; and iteration number, 50000. The MCMC samples were judged to have converged using the Gelman-Rubin statistic (Rhat<1.1), with no drift in all chains.

The mean of the posterior distribution for $\mu_{i_{n},j_{n},k}$ was calculated and organized as a new variable ${\hat{\mu }}_{i_{n},j_{n},k}$ corresponding to all conditions after the MCMC samples had converged. Each element of ${\hat{\mu }}_{i_{n},j_{n},k}$ was defined as the standardized optimal WL/WW (soWL/soWW) for each condition.

### 2.5 Prediction of the optimal windowing parameters

The optimal windowing parameters for a new MR image were predicted using the following procedure. The imaging condition $j_{n}$ of the input MR image was assessed, the MRI operator $i_{n}$ was selected, and the ${\hat{\mu }}_{i_{n},j_{n},k}$ corresponding to this condition was selected. Subsequently, ${\hat{\mu }}_{i_{n},j_{n},k}$ was mapped on the original scale of the input MR image using an inverse function of the mapping function created in the preprocessing step (Fig. 2). As in the preprocessing step, this inverse function was created using the landmark values in the IOIs of the input MR image. The standardized optimal UW and LW (soUW/soLW), calculated from the soWL/soWW using equations equivalent to equations (1), (2), were then input. The optimal UW/LW (oUW/oLW) was output from the inverse function and converted to oWL/oWW using equations equivalent to equations (3), (4).

### 2.6 Qualitative evaluation

oWL/oWW for each MRI operator was predicted, as mentioned above, by the statistical model using some of the imaging conditions in the test set. MR images with the output oWL/oWW and WL/WW of the original values were then displayed for qualitative comparison.

### 2.7 Quantitative evaluation

The prediction performance was evaluated by assessing the mean relative error (MRE), mean absolute error (MAE), and Pearson's correlation coefficient (ρ) as evaluation metrics of the test set [3]. These metrics are defined in equations (8), (9), (10):

(8) $$MRE=\frac{1}{T}\sum_{t=1}^{T}\left|\frac{{\hat{y}}_{t}-y_{t}}{y_{t}}\right|\times 100\text{,}$$

(9) $$MAE=\frac{1}{T}\sum_{t=1}^{T}\left|{\hat{y}}_{t}-y_{t}\right|\text{,}$$

(10) $$\rho =\frac{\sigma_{\hat{y}y}}{\sigma_{\hat{y}}\sigma_{y}}$$

where t is the index of the test set; T is the total number of the test set; ${\hat{y}}_{t}$ and $y_{t}$ are the predicted oWL or oWW and original WL or WW in the test set, respectively; and $\sigma_{\hat{y}y}$ is the covariance between ${\hat{y}}_{t}$ and $y_{t}$. $\sigma_{\hat{y}}$ and $\sigma_{y}$ are the standard deviations of ${\hat{y}}_{t}$ and $y_{t}$, respectively.

The naive average values of WL/WW for each imaging condition in the training set were also calculated. The method that considers these sets as the optimal values is defined as the naive method. MRE, MAE, and ρ were also calculated for the naive method, and its prediction performance was compared with that of the proposed framework.

### 2.8 Visual assessment

Three MRI operators conducted the visual assessment. Using the test set, MR images with the oWL/oWW predicted by the proposed framework and the original WL/WW of the data were displayed on a monitor under viewing conditions similar to those in clinical practice. Subsequently, a pairwise comparison was conducted using 90 MR images comprising 30 images each of the head, abdomen, and pelvis, which were the most frequently examined body regions.

A visual assessment was also conducted by three radiologists as a two-step assessment. Each radiologist was instructed to select one MR image for each imaging condition that they considered most appropriate using the oWL/oWW predicted by the proposed framework for the three MRI operators. Subsequently, each radiologist compared the MR images in the test set with the oWL/oWW of the preferred MRI operator selected earlier for each imaging condition and the original WL/WW. The viewing conditions and MR images were the same as those used during the visual assessment by the MRI operator. A sign test was performed as a statistical hypothesis test using R, with the significance level set at 0.05.

## 3 Results

### 3.1 Data

The MRI data of 1000 patients were acquired, and the data of 11875 series were analyzed. The body regions included the head, neck, abdomen, pelvis, cervical spine, thoracic spine, lumbar spine, breast, and knee joints. The training and test sets comprised 8315 and 3560 images (at a 7:3 ratio), respectively.

### 3.2 Qualitative evaluation

The MCMC samples were judged to have converged as all Rhats were smaller than 1.1, and none of the chains had drifted. Only the representative results, especially those of the head, abdomen, and pelvic region, are presented here, as presenting the entire data is a formidable task.

**Fig. 3:**
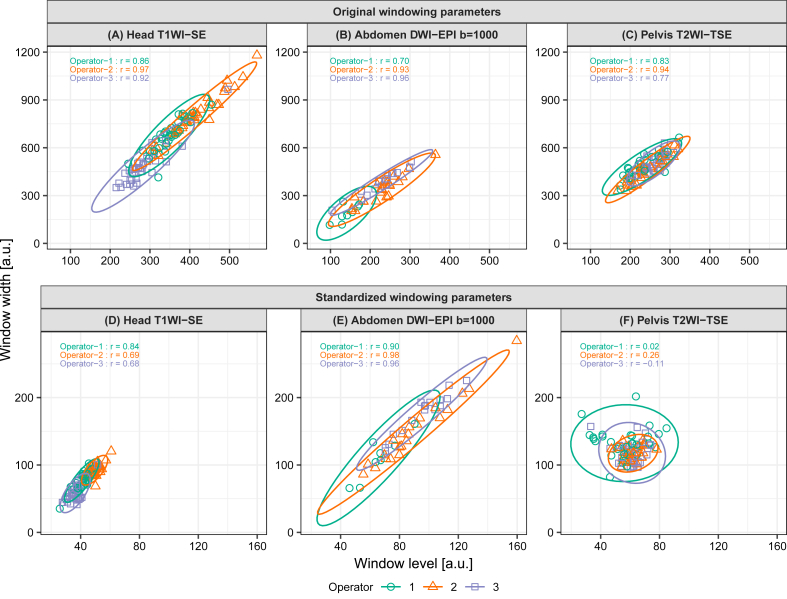
The scatterplots and 95% confidence ellipses of the window level and width (WL/WW) of representative MR images in the training set. The top row (A–C) shows the original WL/WW, whereas the bottom row (D–F) shows the standardized WL/WW. The acquisition sequence and body region are brain spin echo T1 weighted image (T1WI-SE) in the first column (A, D), abdomen diffusion-weighted image with echo planer imaging (DWI-EPI) with b = 1000 in the second column (B, E), and pelvis turbo spin echo T2 weighted image (T2WI-TSE) in the third column (C, F), respectively. The upper left corner of each figure shows the Pearson's correlation coefficients corresponding to the data from MRI operators 1, 2, and 3.

Fig. 3 presents the scatter plots of the WL/WW and sWL/sWW sets for each MRI operator in the training set and the estimated 95% confidence ellipses. The WL/WW and sWL/sWW scatter plots followed a bivariate normal distribution. The pelvic turbo spin echo T2-weighted image (T2WI-TSE) revealed a significant decrease in the correlation coefficient after standardization, suggesting that windowing parameter standardization could alter the relationship between WL and WW.

**Fig. 4:**
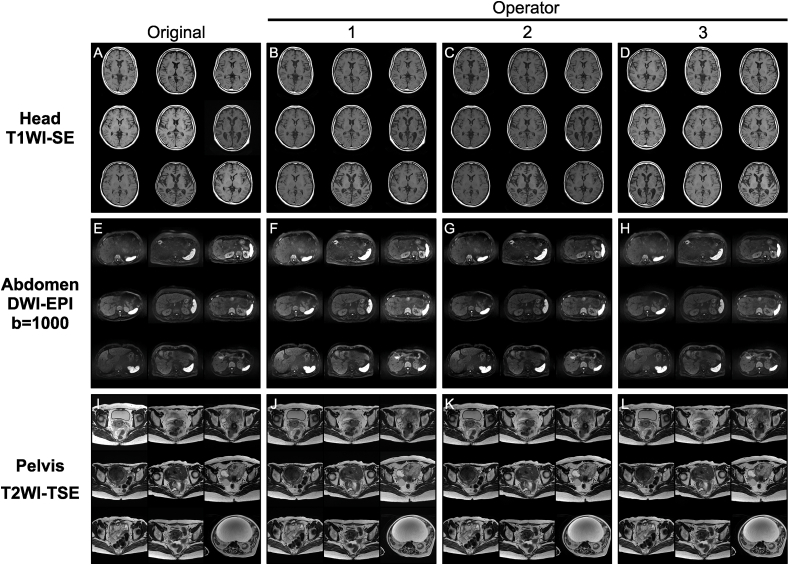
Representative magnetic resonance (MR) images of the test set displayed with the original window level and width (WL/WW; A, E, I) and the predicted optimal WL/WW for each MRI operator (B–D, F–H, J–L). The acquisition sequence and body region are shown for brain spin echo T1-weighted image (T1WI-SE) in the first row (A–D), abdomen diffusion-weighted image with echo planer imaging (DWI-EPI) with b = 1000 in the second row (E–H), and pelvis turbo spin echo T2-weighted image (T2WI-TSE) in the third row (I–L), respectively.

Fig. 4 presents MR images with their original WL/WW and the oWL/oWW predicted by the proposed framework using the test set. The original MR images had non-uniform brightness as they comprised a mixture of the WL/WW data of multiple MRI operators in clinical practice. In contrast, the images with oWL/oWW for each MRI operator predicted by the proposed framework had uniform brightness.

### 3.3 Quantitative evaluation

Compared with the naive method, the proposed framework had average MREs and MAEs that were 6 and 4.8 points and 15.9 and 20.6 lower for WL and WW, respectively (Table 2). However, the correlation coefficients were almost equivalent for both methods. Thus, the proposed framework predicted the windowing parameters better than the naive estimation method.

**Table 2:** Quantitative evaluation of the naive estimation method and proposed framework using the test set data.

		Window level			Window width		
		MRE (%)	MAE	ρ	MRE (%)	MAE	ρ
Naive estimation method	Operator-1	21.9	58.6	0.97	21.4	111.0	0.97
	Operator-2	15.4	58.9	0.98	15.7	98.2	0.98
	Operator-3	18.6	59.0	0.98	18.9	109.0	0.98
	Average	18.6	58.8	0.98	18.7	106.0	0.98
Proposed framework	Operator-1	13.4	41.6	0.98	14.3	83.9	0.98
	Operator-2	12.1	43.0	0.99	13.2	82.5	0.99
	Operator-3	12.5	44.0	0.99	14.0	90.0	0.98
	Average	12.6	42.9	0.98	13.9	85.4	0.98

Abbreviation: MRE, mean relative error; MAE, mean absolute error; ρ, Pearson's correlation coefficient.

### 3.4 Visual assessment

No significant difference in visual preference was observed between the MR images with oWL/oWW predicted by the proposed framework and those with the WL/WW set from clinical practice during the visual assessments conducted by the MRI operators and radiologists (Table 3).

**Table 3:** Percentage of MR images with windowing parameters estimated by the proposed method and the results of the sign test in the visual evaluation.

	**Percentage**	**95%CI**	***P*-value**
Operator-1	0.42	0.32, 0.53	0.17
Operator-2	0.43	0.33, 0.54	0.25
Operator-3	0.44	0.34, 0.55	0.34
Radiologist-1	0.56	0.45, 0.66	0.34
Radiologist-2	0.52	0.41, 0.63	0.75
Radiologist-3	0.46	0.35, 0.56	0.46

Abbreviation: MR, magnetic resonance; CI, confidence interval.

## 4 Discussion

Compared with the naive method, the proposed framework showed superior performance in the quantitative evaluation and could predict the windowing parameters set by the three MRI operators in clinical practice. Furthermore, there was no significant difference in the visual preference between the MR images adjusted to the WL/WW selected by each MRI operator and those predicted by the proposed framework. The same results were obtained by the radiologists. These findings demonstrate that the proposed framework can predict the preferred windowing parameters for each observer under specific viewing conditions.

Adjusting the display conditions of MR images on a monitor is essential for diagnostic imaging. Previous reports have suggested that adjusting the display conditions improves the delineation of focal liver lesions on Gd-EOB-DTPA-weighted MR images [25] and differentiation between benign tumors and cysts of the oral and maxillofacial region on T2-weighted images [26]. Similarly, standardization of the display conditions of diffusion-weighted images has been shown to improve the diagnostic accuracy of insular ribbons and reduce reading time [27]. Although there are many advantages to displaying MR images under proper viewing conditions, this has not yet been realized in clinical practice. One reason for this is that the automatic windowing systems reported in previous studies [[2], [3], [4], [5]], although effective, were not feasible in clinical practice as it was laborious to create such models. The framework proposed in this study utilizes only the windowing parameters set in clinical practice, implying that MR images with appropriate display conditions for each observer can be obtained by simply selecting the best images. In other words, our framework can overcome the limitations of previous models as our framework makes it easier to create models and robustly predicts appropriate windowing parameters.

The model proposed in this study demonstrated mixed results compared with the recently reported CNN model [3]. Although it underperformed in predicting the WL/WW (MAE was 7–9 points lower) and WL (MAE was 6 points lower), it outperformed the CNN model in predicting WW (MAE was 12 points higher) (Table 2). Furthermore, the correlation coefficient ρ improved by approximately 0.3 in the proposed model. However, interpreting these differences is challenging due to the varying nature of the models, datasets, and dataset preparation methods. Nonetheless, visual evaluations revealed that the proposed method could provide suitable display conditions (Table 3), suggesting that a prediction performance equivalent to the results in this study would provide substantially optimal display conditions. Moreover, the dataset of the CNN model consisted of WL/WW values that were determined by multiple clinicians [3], which may have led to individual observer preferences being neglected and considerable effort being required to create the model. In contrast, our framework considers the preferences of each observer and predicts optimal values using existing data or a selection of dataset combinations. This characteristic imparts a significant advantage to our framework in terms of the simplicity of constructing a predictive model.

The robust prediction performance achieved by the proposed model using only the windowing parameters set in clinical practice may be attributed to two reasons. First, the standardization of the windowing parameters was effective. The image intensity standardization method proposed by Nyul et al. [6,7] is often used in machine-learning models that use MR images [15,17,[19], [20], [21]]. These machine learning models, which use this standardization method for preprocessing, have shown good performance, suggesting that this standardization method can robustly standardize the signal intensity of MR images. Fig. 3 demonstrates that the distribution of the windowing parameters changes after standardization, indicating that the windowing parameters are effectively standardized. Thus, this method may be effective for predicting the windowing parameters corresponding to the signal intensity values employed in this study.

Second, the assumptions of the statistical model used in this study were valid. The windowing parameters are adjusted such that the MR images of the same acquisition sequence and body region are displayed similarly on the monitor of each observer. However, variations may be present in the values as human beings do not have perfect reproducibility. It is common to assume that such noise is Gaussian; hence, the assumption in this study was that the windowing parameters after standardization follow a bivariate normal distribution. As shown in Fig. 3, the windowing parameters for each imaging condition approximately followed a bivariate normal distribution after standardization. Furthermore, the assumption of the mixed-effect model for the mean vector resulted in an appropriate representation of the differences in the acquisition sequence, body region, and MRI operator, contributing to improved prediction performance. Although estimating the parameters of complex models, such as the proposed model, has conventionally been challenging, the Bayesian statistical framework enables parameter estimation through numerical calculations [28]. Therefore, it is believed that high prediction performance can be achieved by adjusting the windowing parameters in clinical practice.

The results of the visual assessment also support the usefulness of the proposed framework. The MRI operators reported no differences that were visually significant between the MR images displayed with the windowing parameters adjusted by themselves and those displayed with the predicted parameters. Although the visual assessment conducted by the radiologists showed no visually significant difference, the results should be interpreted with caution. The radiologists selected one of the WL/WW set by the three MRI operators as a reference for those predicted by the proposed framework. Thus, they may have been forced to select one of the values even if the viewing conditions were undesirable, as the reference windowing parameters were not adjusted by them. As a result, the MR images displayed with both the reference windowing parameters and the predicted windowing parameters would have been unfavorable, and it is possible that there was no significant difference. However, since the radiologists and MRI operators in this study worked at the same facility, the MRI operator are more likely to present MR images with display conditions that are favored by the radiologist. Regular discussions are held between the MRI operators and radiologists to ensure that the display conditions of the MR images are generally acceptable to the WL/WW in each radiologist's displaying conditions at the facility from where the data were obtained in this study. Therefore, it is likely that there was no significant difference as MR images displayed with the predicted and reference windowing parameters were equally favorable. The proposed framework is expected to successfully provide MR images with proper display conditions and minimal image selection effort as a preliminary preparation, even for radiologists who require the adjustment of the windowing parameters in clinical practice.

A limitation of this study is that only MR images from a single manufacturer at a single facility were used. Since the distribution of pixel intensities in MR images differs from scanner to scanner [8], the model must be extended to adapt this framework to data from multiple manufacturers and facilities. This limitation could be addressed by adding parameters to the statistical model representing differences between the manufacturers and facilities. Future studies should include more diverse data in the model. Second, the MR images used in this study were only used for acquisition sequences and body regions that are frequently scanned in routine clinical settings. This study did not propose a method that can be generalized to MR images that were acquired unexpectedly. This is the most crucial challenge in implementing the proposed framework in clinical practice. However, this challenge could be addressed by considering alternative approaches, such as substituting the parameters of the T1-weighted finger image that was acquired unexpectedly with those of a T1-weighted knee image that has already been acquired (i.e., substituting parameters from other images that are expected to have similar signal intensity distributions). If this challenge can be addressed, the proposed framework, which enables convenient model construction and robust prediction of preferred windowing parameters, can be widely adopted by many medical facilities as a windowing parameter predictor that can be easily implemented in clinical practice.

## 5 Conclusions

This study proposed a framework for predicting the windowing parameters that was considered preferable by the observer based on statistical modeling applied to MR images that were standardized using the signal intensity standardization method. The results showed that this framework could predict windowing parameters that were quantitatively and qualitatively close to the display conditions preferred by an observer with high performance. In the future, it is essential to incorporate this framework into clinical practice and verify whether its application would reduce the burden on radiologists and make effective use of resources in diagnostic imaging. The dissemination of this framework in routine clinical practice can lead to increased efficiency of diagnostic imaging and optimized resource allocation.

## Credit author statement

Kohei Sugimoto: Conceptualization, Methodology, Software Data analysis, Visualization, Writing–original draft. Masataka Oita: Supervision, Writing-Review and Editing. Masahiro Kuroda: Supervision, Writing-Review and Editing

## Data availability statement

The source code and the estimated parameters are publicly available on the author's GitHub page: [https://github.com/SugimotoKohei/Prediction_Optimal_Windowing_Parameters_in_MRI].

## Funding information

This research did not receive any specific grants from funding agencies in the public, commercial, or not-for-profit sectors.

## Declaration of competing interest

The authors declare that they have no known competing financial interests or personal relationships that could have appeared to influence the work reported in this paper.
